# Diabetes and cardiovascular disease: from evidence to clinical practice – position statement 2014 of Brazilian Diabetes Society

**DOI:** 10.1186/1758-5996-6-58

**Published:** 2014-05-20

**Authors:** Marcello Casaccia Bertoluci, Augusto Pimazoni-Netto, Antonio Carlos Pires, Antonio Eduardo Pesaro, Beatriz D Schaan, Bruno Caramelli, Carisi Anne Polanczyk, Carlos Vicente Serrano Júnior, Danielle M Gualandro, Domingos Augusto Malerbi, Emilio Moriguchi, Flavio Antonio de Oliveira Borelli, João Eduardo Nunes Salles, José Mariani Júnior, Luis Eduardo Rohde, Luis H Canani, Luiz Antonio Machado Cesar, Marcos Tambascia, Maria Tereza Zanella, Miguel Gus, Rafael Selbach Scheffel, Raul Dias dos Santos

**Affiliations:** 1 Faculdade de Medicina da Universidade Federal do Rio Grande do Sul, Porto Alegre, Brazil; 2 Universidade Federal de São Paulo, São Paulo, Brazil; 3 Faculdade Estadual de Medicina de São José do Rio Preto, São Paulo, Brazil; 4 Hospital Albert Einstein, São Paulo, Brazil; 5 Faculdade de Medicina da Universidade de São Paulo, São Paulo, Brazil; 6 Sociedade Brasileira de Cardiologia, São Paulo, Brazil; 7 Sociedade Brasileira de Diabetes, São Paulo, Brazil; 8 Universidade de São Paulo, São Paulo, Brazil; 9 Faculdade de Ciências Médicas da Santa Casa de São Paulo, São Paulo, Brazil; 10 Faculdade de Ciências Médicas da Universidade de Campinas, Campinas, SP, Brazil; 11 Hospital de Clínicas de Porto Alegre, Porto Alegre, RS, Brazil

**Keywords:** Diabetes, Glicemic control, Cardiovascular risk stratification, Screening coronary artery disease, Treatment of dyslipidemia, Hypertension, Antiplatelet therapy, Myocardial revascularization

## Abstract

There is a very well known correlation between diabetes and cardiovascular disease but many health care professionals are just concerned with glycemic control, ignoring the paramount importance of controlling other risk factors involved in the pathogenesis of serious cardiovascular diseases. This Position Statement from the Brazilian Diabetes Society was developed to promote increased awareness in relation to six crucial topics dealing with diabetes and cardiovascular disease: Glicemic Control, Cardiovascular Risk Stratification and Screening Coronary Artery Disease, Treatment of Dyslipidemia, Hypertension, Antiplatelet Therapy and Myocardial Revascularization. The issue of what would be the best algorithm for the use of statins in diabetic patients received a special attention and a new Brazilian algorithm was developed by our editorial committee. This document contains 38 recommendations which were classified by their levels of evidence (A, B, C and D). The Editorial Committee included 22 specialists with recognized expertise in diabetes and cardiology.

## Introduction

This Position Statement is a review based on the best currently available scientific evidence to guide the prevention and management of cardiovascular disease in patients with diabetes. The document was developed by endocrinologists and cardiologists gathered in the Group of Experts on Diabetes and Cardiovascular Risk, which contributed significantly to the development of this document, formatted into practical recommendations regarding management of cardiovascular disease in these patients.

This paper innovates by reviewing the concept of equivalence of cardiovascular risk in patients with diabetes in light of recent evidences suggesting the presence of lower-risk subgroups within this population. On the other hand, targets of LDL-c on treatment with statins are no longer recommended and the suggestion to make therapeutic decisions based on the stratification of cardiovascular risk by use of the calculator UKPDS cardiovascular risk assessment is now enforced.

Also incorporated, now in a more defined way, the use of calcium score in more specific situations regarding the use of statins. It was also included an update on the recommendations for the treatment of hypertension, for the use of antiplatelet and finally an update on indications for myocardial revascularization exclusively in patients with diabetes.

The Brazilian Diabetes Society (SBD = Sociedade Brasileira de Diabetes) hopes that this review will be useful in clinical practice targeting an increased quality of care for patients with diabetes.

## Methodology

This Position Statement rated levels of evidence according to Table 1, and for each recommendation, the evidence is presented below.

**Table 1:** Levels and descriptions of evidence

**Levels of evidence**	**Description**
Level A	*Direct evidence from meta-analysis or randomized clinical trials*
Level B	*Evidence from large observational studies and indirect evidence analysis of pre-specified subgroup of randomized clinical trials or meta-analysis with low heterogeneity.*
Level C	*Evidence from small studies, non-randomized or open*
Level D	*Expert opinion*

Originally, the members of the editorial board defined the topics considered relevant and that required positioning by SBD. Thereafter a search of the literature was performed to select the most important studies. It was then drafted a preliminary manuscript with the respective levels of evidence which underwent several rounds of discussion among committee members for review and suggestions. The manuscript then returned to the chief editor for tweaking and unifying editorial style. The manuscript was subsequently subjected to further revisional rounds by some members of the committee in seeking a consensus position and after this phase it was forwarded for final editoring and then submitted for publication.

### Recommendations

The recommendations of this Position Statement of the Brazilian Diabetes Society will be divided into six modules, namely:

– **Module 1:** Glicemic Control

– **Module 2:** Cardiovascular Risk Stratification and Coronary Disease Screening

– **Module 3:** Treatment of Dyslipidemia

– **Module 4:** Treatment of Hypertension

– **Module 5:** Antiplatelet Therapy

– **Módulo 6:** Myocardial Revascularization

– **References**

The numbered references in the texts of each module are described at the end of the full text.

### Module 1: glycemic control

1. ***In adult patients with diabetes mellitus, target HbA1c of approximately 7.0% is recommended. [Level A]***

Summary of evidence:

• *The DCCT*[1]*and UKPDS*[2,3]*classic studies conducted in subjects with type 1 and type 2 diabetes have demonstrated convincingly that intensive glycemic control (HbA1c ~ 7.0%) reduces chronic microvascular complications*[4]*and, in the long- term, can also reduce the occurrence of non-fatal acute myocardial infarction*[5,6]*. Evidence from 3 large clinical trials in patients with type 2 diabetes - ADVANCE*[7]*, ACCORD*[8]*and VADT*[9]*, which evaluated more-intensive compared to less- intensive glycemic targets, showed only reduced microvascular, but not macrovascular complications. Meta-analysis that included these studies showed marginal reduction in the risk of myocardial infarction with more intensive glycemic control, but with an increase in the number of severe hypoglycemias*[10]*. Thus, the target for the treatment of hyperglycemia in type 2 diabetes is to achieve A1C levels of ~ 7%, and specific glycemic goals are: fasting glucose between 70–130 mg/dL and postprandial glucose <180 mg/dL*[11]*.*

2. ***In elderly patients with co-morbidities that significantly limit life expectancy, in whom the risk of hypoglycemia is even more harmful, it is acceptable to seek an A1C target level of up to 7.9%. [Level B]***

Summary of evidence:

• *Less stringent target (A1C 7.0 to 7.9%) should be considered in patients with a history of frequent episodes of hypoglycemia, late-onset diabetes, micro or macrovascular advanced disease or when there is difficulty in maintaining good glycemic control, despite the association of various medicines. In ACCORD*[8]*study, although there was a reduction in cardiovascular morbidity, there was also an increase in mortality with more intensive control. A meta-analysis showed only marginal reduction in the risk of myocardial infarction with intensive glucose control, but with an increase in the number of severe hypoglycemia*[10]*. Data from observational studies also reinforce the need to target A1C to less stringent levels, showing that the lowest risk of mortality occurs around an A1C of 7.5%*[12]*. The absolute benefit obtained with intensive treatments in 5 years is modest: the number needed to treat (NNT) to prevent one event is 140 for ischemic heart disease, 768 for stroke, 272 for mono-ocular blindness and 627 for ESRD. In turn, the number needed to harm (NNH) is 328 for total mortality and 21 for severe hypoglycemia*[10,13]*. Furthermore, the maintenance of A1C from values below 8% is also less cost-effective*[14]*.*

3. ***In patients hospitalized for acute myocardial infarction (AMI), it is suggested to maintain blood glucose between 130 and 180–200 mg/dL using continuous intravenous insulin. [LEVEL B]***

Summary of evidence:

• *Three randomized clinical trials have evaluated the role of glycemic control in the incidence of cardiovascular events after an acute myocardial infarction (AMI) in patients with diabetes. The first was the DIGAMI*[15]*study, in which 620 patients with DM and AMI were included. Treatment strategies were: infusion of insulin and glucose IV in the first 24 hours with a glycemic target of 126–196 mg/dL, followed by subcutaneous administration of insulin four times daily for 3 months vs. insulin therapy only when clinically indicated. The group using insulin in the acute phase had better glycemic control during hospitalizationat 3 months and at 1 year, and also had lower mortality rates at 1 and 3.4 years of follow-up.*

• *The DIGAMI-2*[16]*compared 3 groups: insulin during hospitalization followed by outpatient use, use of insulin only during hospitalization and usual treatment throughout the period. Glycemic control was similar between groups as well as cardiovascular outcomes.*

• *HI-5*[17]*study included 240 patients with a history of diabetes and glucose ≥ 140 mg/dL on hospital admission for AMI, who were randomized to strict glycemic control (target 72–180 mg/dL) with insulin plus intravenous glucose infusion for at least 24 hours or conventional therapy. Subsequently, patients were managed by their physician, with a recommendation to maintain A1C < 7%. The rates of in-hospital mortality did not differ between groups.*

4. ***In patients in the immediate postoperative cardiac surgery, it is recommended to maintain blood glucose between 120 and 150 mg/dL using continuous intravenous insulin. [LEVEL A]***

Summary of evidence:

• *Hyperglycemia before or after cardiac surgery is associated with a higher risk of complications such as death, prolonged mechanical ventilation, renal failure, stroke and deep sternal infection*[18,19]*.*

• *The Portland Diabetes Project was an observational study that evaluated the relationship between hyperglycemia and adverse outcomes of cardiac surgery in patients with diabetes. It consisted of using continuous intravenous insulin, adjusted by frequent blood glucose tests based on standardized protocol conducted by nurses, with target glycemia 150–200 mg/dL. Subsequently, this blood glucose target has changed to 125–175 mg/dL and then to 100 to 150 mg/dL, because studies in other scenarios were identifying the need for normalization of blood glucose reduction outcomes. The use of this protocol compared with the use of subcutaneous insulin according to glucose levels (historical control) was associated with reduced rates of infection*[20]*and death in about 50%*[21]*.*

• *A randomized clinical trial with patients in the surgical intensive coronary unit (ICU), (mostly in post-cardiac surgery (63%) and 13% with diabetes) showed benefit with intensive glycemic control (insulin infusion for glycemic target 80–110 mg/dL vs. 180–200 mg/dL) in mortality, infection, acute renal failure with hemodialysis, blood transfusion, and polyneuropathy in critically ill patients, but at the expense of higher rates of hypoglycemia*[22]*.*

• *However, multicenter clinical trial with larger number of patients (Nice Sugar Study) conducted in medical and surgical ICUs (63% and 37% of patients, respectively), 20% with a history of diabetes, showed that intensive glycemic control (target <108 mg/dL) vs. usual control (140 to 180 mg/dL) resulted in increased mortality and also higher rates of hypoglycemia*[23]*.*

• *Meta-analysis including data from NICE SUGAR study, evaluating separately results obtained from clinical and surgical ICUs, showed that tight glucose control offers no reduction of mortality in patients in clinical ICU, but may have benefit in surgical patients when target blood glucose is below 150 mg/dL*[24]*. In a small randomized clinical trial comparing two recent glycemic targets (90–120 mg/dL vs. 120–180 mg/dL) in patients with diabetes undergoing coronary artery bypass grafting, a more stricted blood glucose control provided no benefit and even increased the risk of hypoglycemia*[25]*.*

### Module 2: cardiovascular risk stratification and screening of coronary artery disease

5. ***It is recommended that patients with type 2 diabetes without a history of cardiovascular disease have their cardiovascular risk stratified annually by the UKPDS risk-calculator, using the outcome: (CHD - Coronary Heart Disease) in 10 years. Through this tool, patients should be divided into low risk (<10% in 10 years), intermediate risk (10-20% in 10 years) and high risk (>20% in 10 years). [LEVEL D]***

Summary of evidence:

• *The UKPDS calculator was validated from a multiethnic population of the United Kingdom, originating from the UKPDS study, with 4,540 participants with type 2 diabetes between 25–65 years of age without a prior history of myocardial infarction, angina or heart failure*[26]*. The calculator has its best performance in the range of intermediate risk, with low levels of underestimation (13%), being higher than the overall risk scores developed for the general population like the Framingham score, if applied to a population with diabetes*[27]*.*

**Figure 1:**
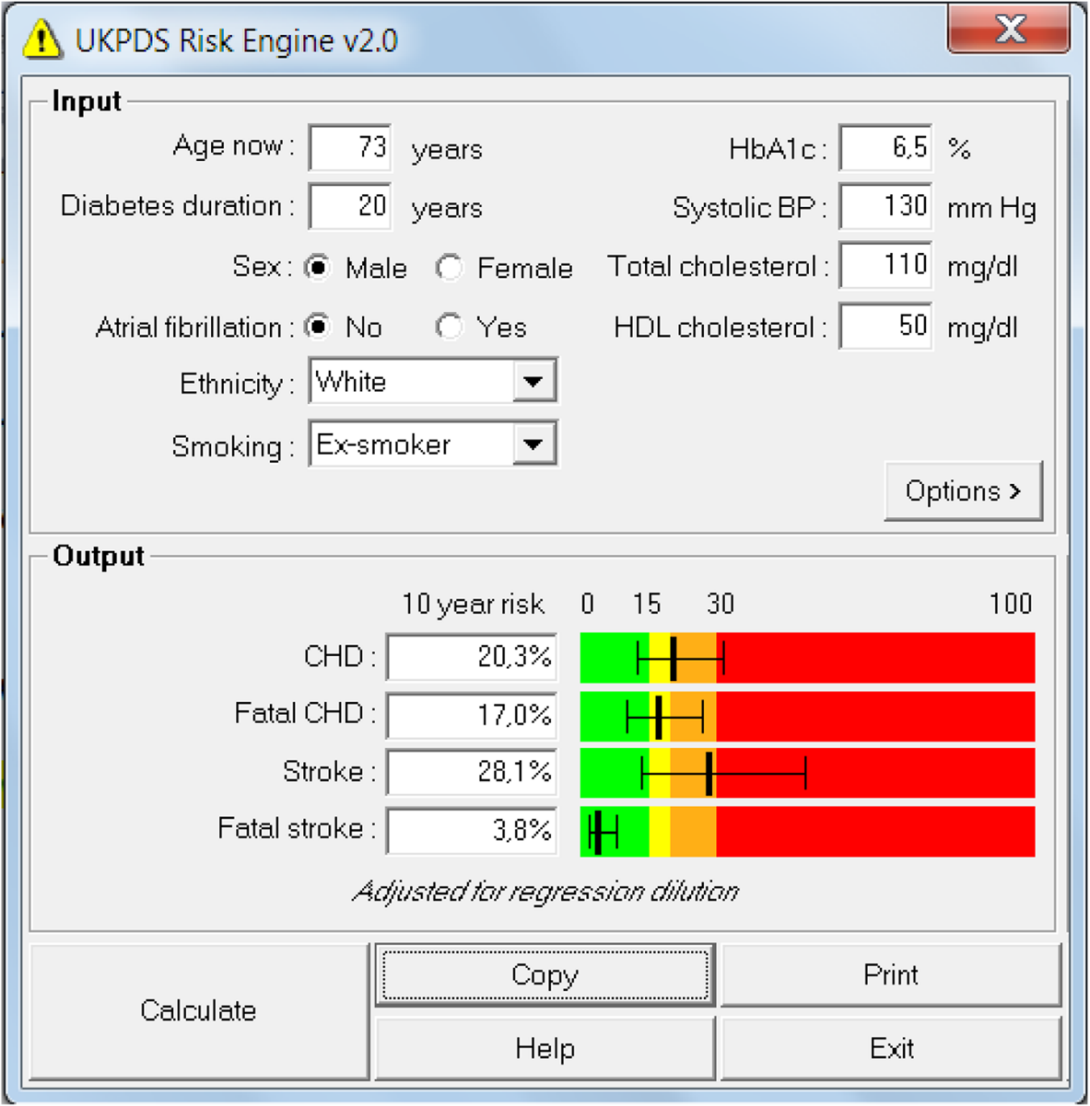
Example of calculating cardiovascular risk with the use of a UKPDS calculator, considering the parameters provided.

*Figure*1*shows the screen of the UKPDS calculator for the assessment of cardiovascular risk, taking into account the following parameters: age, duration of diabetes, sex, presence or absence of atrial fibrillation, ethnicity, presence or absence of smoking, A1C level, systolic blood pressure, total cholesterol and HDL cholesterol. With the inclusion of these data by the operator, this tool calculates the 10-year risk of occurrence of fatal and nonfatal CHD and fatal and nonfatal stroke* (Figure 1)*.*

This calculator can be downloaded without charge from the following address:

http://www.dtu.ox.ac.uk/riskengine/download.php

6. ***In patients with diabetes and cardiovascular risk assessed as an intermediary by the UKPDS calculator, we suggest performing the evaluation of coronary calcium score (CAC) in order to reclassify to low risk when the score is less than 10 or intermediate/high risk when more than 10, when available. [LEVEL A]***

Summary of evidence:

• *The coronary artery score (CAC) proved to be a good identifier of low-risk sub-population of patients with diabetes. A meta-analysis of 8 observational studies in 6,521 patients with diabetes with a mean follow-up of 5.2 years, comparing CAC <10 vs. >10, showed a relative risk of death from all causes or cardiovascular events in the group >10, 5.47 times higher (95% CI 2.59 to 11.53). For patients with CAC < 10, post-test probability of a primary endpoint was 1.8%, representing a reduction of 6.6 times from the pre-test probability. The CAC <10 result was present in 28.5% of patients with diabetes. Given the global prevalence of diabetes of 346 million, these data suggest that 86.5 million people with diabetes may have low cardiovascular risk*[28]*. A re-analysis of Jupiter and the MESA study*[29]*showed that in the general population, CAC = 0 corresponds to a cardiovascular event rate of 0.8 per 1000 person-years.*

7. ***It is recommended to conduct an annual electrocardiogram for screening of coronary heart disease in patients with type 2 diabetes. [LEVEL D]***

8. ***In asymptomatic diabetic patients under 75 years old, without rest ECG abnormalities and without previous positive stress testing, it is recommended not to perform universal screening tests for coronary disease with induction of myocardial ischemia. [LEVEL A]***

Summary of evidence:

• *The Detection of Ischemia in Asymptomatic Diabetics (DIAD)*[30]*evaluated 1,123 participants with type 2 diabetes without symptoms of coronary artery disease, randomized to screening with induction of ischemia test (myocardial perfusion adenosine stress (n = 562) or no screening (n = 561). Patients with evidence of cardiovascular disease or changes in the resting electrocardiogram were excluded from the protocol. Patients with a mean age of 61 years, duration of diabetes > 8 years and mean A1C of 7.1% were included. The cumulative rate of cardiac events (nonfatal myocardial infarction and cardiovascular death) was lower than expected, occurring in 2.9% of patients in the follow-up of 4.8 years. In total, only 15 cardiac events occurred among the screened patients and 17 among non- screened (OR 0.88, 95% CI 0.44 to 1.88, p = 0.73). While screening has not implicated in a significant difference in clinical outcomes, patients with moderate to large myocardial perfusion defects had higher rates of cardiac events (OR 6.3, 95% CI 1.9 to 20.1, p = 0.001). A sub-analysis of 5 years following this same study*[31]*has suggested that the majority of enrolled patients were classified as intermediate or high cardiovascular risk. However, the annual risk of cardiac events in these patients remained low and was not altered by the strategy of screening for inducible ischemia, so the conclusion is that the screening of myocardial ischemia does not reduce the rate of cardiac events in patients with asymptomatic diabetes.*

9. ***It is recommended to assess myocardial ischemia in patients with cardiovascular symptoms or with evidence of atherosclerotic disease in other vascular sites (peripheral vascular disease, carotid bruit, transient ischemic stroke or previous episodes, or presence of Q waves on resting ECG). [LEVEL D]***

10. *** The treadmill stress test is recommended as initial test for most patients who present indication for the investigation, excepting those who are unable to walk, those with resting ECG showing changes that hinder the interpretation of the test, or those with contraindications for the test. [LEVEL D]***

### Module 3: treatment of dyslipidemia

11. *** Patients with diabetes and a prior history of cardiovascular events (myocardial infarction, stroke, coronary revascularization, atherosclerotic disease of carotid, renal or peripheral arteries and the aorta) should begin intensive treatment with statins. [LEVEL A]***

Summary of evidence:

• *Statins have proven to reduce the risk of cardiovascular events in patients with diabetes and a previous history of vascular events. A meta-analysis of 14 trials including 18,686 patients with diabetes showed that treatment with statins reduces proportionately the incidence of vascular events by 20% for each 39 mg/dL reduction of LDL-c in 5 years and the reductions are similar for major coronary events, stroke and need for revascularization*[32]*.*

12. *** In patients with diabetes and confirmed cardiovascular disease, it is recommended to reduce LDL-c at least 50% of the baseline values with statins at the highest tolerated doses. Alternatively it is reasonable to reduce LDL-c below 70 mg/dL with statins. [LEVEL B]***

Summary of evidence:

• *A comparison between intensive vs moderate statin treatment was evaluated in a meta-analysis of 5 randomized trials with 39,612 individuals, where 5,639 (19%) had diabetes, and 59% had prior vascular disease, with a mean follow-up of 5.1 years*[33]*. Intensive treatment was defined as a reduction of 20 mg/dL LDL-c beyond the result obtained by moderate treatment with the use of higher power statins. The results showed 15% reduction in major vascular events (95% CI 11–18, p < 0.0001), 13% in coronary death (95% CI 7–19, p < 0.0001), 19% in coronary artery myocardial (95% CI 15–24 p < 0.0001) and 16% in stroke (95% CI 5–26, p = 0.005). Intensive treatment promoted a 20% reduction in LDL-c beyond the moderate treatment, while the moderate treatment promoted a decrease of 30% compared to placebo. Thus, there was an overall 50% reduction in events with intensive treatment compared to placebo. Although this is an indirect evidence, based in subgroup analysis of diabetic patients included in the meta-analysis, the absence of heterogeneity makes it plausible to be applied to patients with DM in secondary prevention.*

• *A pre-specified subgroup analysis of the Treat to New Targets (TNT) study, which included 1,501 patients with diabetes and coronary artery disease*[34]*, compared the treatment with atorvastatin 80 mg vs. atorvastatin 10 mg for 4.9 years in cardiovascular outcomes. The study showed a significant reduction in any cardiovascular events and strokes in patients with a dose of 80 mg. Patients taking 10 mg maintained an average level of LDL-c of 96 mg/dL, while those using 80 mg/day reached 77 mg/dL. Thus, the use of a target LDL-c around 70 mg/dL showed additional benefit. This committee considered that obtaining the target of 70 mg/dL is quite similar to a 50% reduction in baseline LDL-C in the great majority of cases, and therefore may alternatively be used as a target to facilitate treatment adherence.*

13. *** It is recommended that patients with LDL-c > 190 mg/dL receive statin treatment regardless of having or not a previous cardiovascular event, with the goal of a 50% reduction in cholesterol levels. [LEVEL D]***

Summary of evidence:

• *Although most of the clinical trials do not include patients with LDL above 190 mg/dL, indirect evidence from several clinical trials shows that every 39 mg/dL reduction in LDL-c produces a reduction of 20% in the risk of events related to cardiovascular atherosclerotic disease*[35]*. This positioning is in accordance to the ACC/AHA 2013 guidelines that recommends to use statins due to the high probability of associated familial hypercholesterolemia*[36]*.*

14. *** It is recommended that dialysis patients do not initiate the use of statins for lack of evidence of benefit in this population, with possible increase in risk of stroke. However, it is recommended not to withdraw the statin in patients with chronic renal failure who are already in use of statin before initiation of dialysis. [LEVEL A]***

Summary of evidence:

• *The 4D study*[37]*evaluated 1,255 patients with type 2 diabetes on hemodialysis that were randomized to atorvastatin 20 mg or placebo and followed up for 4 years. The primary endpoint was a composite of death from cardiac causes, nonfatal myocardial infarction, and stroke. There was a 42% reduction in LDL-c in patients using atorvastatin, however there was no reduction in the primary outcome, and yet it increased the risk of stroke in this group.*

• *The AURORA study*[38]*was a randomized, multicenter trial which included 2,776 hemodialysis patients, with ages between 50–80 years, being 27.9% with diabetes which were treated with rosuvastatin 10 mg/day or placebo during a mean of 3.8 years. The evaluated primary outcome was a composite of nonfatal myocardial infarction, nonfatal stroke, and cardiovascular death. Even with a 43% reduction in LDL-c in the intervention group, no differences in the primary outcome were observed between groups.*

• *In respect of patients with chronic renal disease but not in hemodialysis, an analysis of the database of Pravastatin Pooling Project combined results of 3 randomized trials using pravastatin 40 mg vs. placebo*[39]*including 19,700 patients with chronic renal insufficiency (GFR 60–30 ml/min/1, 73 m*^2^). *This analysis showed significant benefit of treatment in reducing the primary end point of myocardial infarction, coronary death or percutaneous revascularization and total mortality in this particular group of patients. In light of these findings, there are potential benefits for the use of statins in patients in pre-dialysis, so that the present positioning recommends not to remove the statin in patients who were already in use of statin prior to the start of hemodialysis.*

15. *** It is recommended that patients with heart failure class II to IV do not initiate statin therapy because there is no clear evidence of benefit in this group. [LEVEL A]***

Summary of evidence:

• *The randomized, multicenter clinical trial (GISSI-HF) evaluated rosuvastatin 10 mg/day compared to placebo, in 2,285 patients with heart failure at classes II to IV (New York Heart Association), including 26% of patients with diabetes. There was no benefit over the outcomes: death and hospitalization for cardiovascular causes*[40]*.*

• *The randomized CORONA study, with 5,011 patients over 60 years showing heart failure class II to IV, where 29% had diabetes, compared the use of rosuvastatin 10 mg versus placebo, assessing the primary endpoint composed of cardiovascular death, acute non-fatal MI and non-fatal stroke during 36 months. Even reducing LDL-c in 45% there was no significant difference between the groups regarding the primary outcome. The results were extensive to patients with diabetes in the subgroup analysis due to low heterogeneity*[41]*.*

16. *** Patients with type 2 diabetes without a history of cardiovascular events, aged 40–75 years, with 1 or more risk factors (hypertension, retinopathy, micro or macroalbuminuria, smoking or family history of coronary heart disease) should start treatment with statins. [LEVEL A]***

Summary of evidence:

• *In CARDS study*[42]*, 2,838 patients with diabetes without coronary artery disease prior to age 40 and 75 years and at least 1 additional risk factor (microalbuminuria, retinopathy, hypertension or smoking) were randomized to atorvastatin 10 mg or placebo during a mean follow-up of 3.9 years. The primary outcome was a composite of acute coronary events, coronary revascularization or stroke. The study was terminated prematurely due to efficacy. Atorvastatin 10 mg promoted risk reduction of 37% (95% CI -52 to -17, p = 0.001) in the primary endpoint, a reduction of 32% (95% CI -45 to -15, p = 0.001) in the risk stroke and a trend of 27% reduction in total mortality (95% CI -48 to 1,0 p = 0.059). By this study it is estimated that one event is avoided for every 27 patients treated for 4 years.*

• *The HPS (MRC/BHF Heart Protection Study) substudy*[43]*randomized 5,963 individuals with diabetes aged 40–80 years, to receive simvastatin 40 mg or placebo. Prespecified subgroup analysis was performed for the outcomes of fatal and non-fatal acute myocardial iInfarction (AMI) and the first vascular event (major coronary event, stroke, or revascularization). Simvastatin 40 mg reduced these outcomes in 33% (95% CI 17–46, p < 0.0003), regardless of the level of baseline LDL-c. The absolute risk reduction of cardiovascular disease in patients with diabetes without coronary artery disease in HPS was very similar to the CARDS study, confirming the benefit of statins in patients with diabetes in primary prevention in the high-risk group.*

17. *** Treatment with statins is recommended in patients with cardiovascular risk classified as intermediate or high risk by UKPDS risk engine. [LEVEL B]***

Summary of evidence:

• *A meta-analysis of 22 randomized controlled trials evaluated 134,537 subjects at low cardiovascular risk comparing treatment with statins against placebo or less intensive statin therapy for 4.8 years who were subdivided into strata of cardiovascular risk. In stratum 10-20%/10 years, there were 18% of patients with diabetes. There was a 21% reduction in major vascular events for each 39 mg/dL reduction in LDL-c, regardless of age, gender, baseline LDL-c or presence of previous vascular disease. Considering patients without prior vascular disease with risk between 10-20% in 10 years, there was a 34% reduction in major vascular events for each mmol reduction in LDL-c in 5 years, with no increase in cancer incidence or mortality from other causes. It is estimated that 15 adverse events could be avoided for each 39 mg/dL reduction of LDL-c in 1000 patients treated for 5 years. The benefit was greater than the incidence of adverse events, even in diabetes patients*[44]*.*

18. *** This Position Statement did not find evidences to support the recommendation for treating to LDL-c target of LDL-c < 100 mg/dL in patients with diabetes without established cardiovascular disease.***

*After an extensive review, this board did not find any randomized clinical trial indicating that a titrated drug therapy to a specific LDL-c goal does improve cardiovascular outcomes in diabetic subjects. This recommendation is in accordance to the 2013 ACC/AHA guideline on the treatment of blood cholesterol to reduce atherosclerotic cardiovascular risk in adults*[36]*. Although the American Diabetes Association 2014 position statement still suggests the use of LDL-c targets for patients with diabetes, they also recommend a relative reduction LDL-c of 30-40% from baseline as an alternative effective goal*[45]*. The present Position Statement agrees that the use of LDL-c target of 100 mg/dl may result in under-treatment with statins which have a large body of evidence of event reduction or even overtreatment with non-statin drugs that have no evidence in reducing cardiovascular outcomes despite LDL-c reduction.*

19. *** In patients with intermediate coronary heart risk (10-20% in 10 years) the calcium score (CAC) can be determined, if available. Patients with CAC scores below 10 may be considered of low cardiovascular risk and should be oriented about changes in lifestyle (healthy diet, weight loss if overweight or obesity and physical activity). Patients with calcium score greater than 10 are considered CAC intermediate-high risk and should be treated with statins. [LEVEL A]***

Summary of evidence:

• *Meta-analysis of 8 studies*[28]*including 6,521 patients with diabetes and 5.2 years of follow-up compared mortality and vascular events according to calcium score. Mortality from all causes in patients with CAC score <10 compared to patients with CAC score > 10 was 5.47 times lower (95% CI 2.59 to 11.53, p < 0.001). For patients with CAC score <10, the post-test probability of primary outcome was 1.8%, representing a reduction of 6.8x compared to pre-test probability. The risk of cardiovascular events in patients with a CAC score <10 was 9.22 (95% 2.73 to 31.07 p = 0.005). The prevalence of patients with CAC score <10 was 28.5%. Patients with CAC score above 10 have cardiovascular risk of 3% per year and are considered intermediate to high risk*[28]*.*

20. *** Patients with diabetes and low cardiovascular risk (CHD <10% in 10 years) may receive only treatment with changes in lifestyle (healthy diet, weight loss if overweight/obesity and physical activity), however the coronary risk should be re-evaluated yearly. [LEVEL B]***

Summary of evidence:

• *In the same meta-analysis mentioned in the previous recommendation*[28]*, 24,790 patients were classified as low cardiovascular risk and 7% had diabetes. In this group, the benefit found was a reduction in 6 major cardiovascular events per 1000 patients treated for 5 years for each reduction of 39 mg/dL LDL-c. By the data of this metanalysis, only 1,2-vascular deaths for every 1000 patients treated with statins for 5 years could be avoided with a reduction of LDL-c of 39 mg/dL. This Position Statement held that, although there was no increase in adverse events in 5 years, the benefits are too faint for a systematic indication of statin for this stratum of risk and a clear isk-benefit study is still needed. This position, however, reiterates the need for annual review of risk stratification for possible re-classification.*

21. *** Association between statin and fibrate is not usually recommended for patients with diabetes to reduce cardiovascular risk. However, in the specific situation of men with triglycerides above 204 mg/dL in association with HDL-c below 34 mg/dL, the combination fenofibrate-statin may be considered. [LEVEL B]***

Summary of evidence:

• *This is a recommendation based on the analysis of pre-specified subgroup analysis of patients with diabetes from the ACCORD-LIPID study*[46]*, comparing the fenofibrate-simvastatin combination versus simvastatin alone whose trial showed no reduction in the primary outcome. As there was benefit in prespecified subgroup analysis of men with triglycerides above 204 mg/dL and HDL-C less than 34 mg/dL, this committee considers that the information is still preliminary and should be viewed with caution.*

#### Algorithm SBD-2014 for the use of statins in patients with diabetes

**Figure 2:**
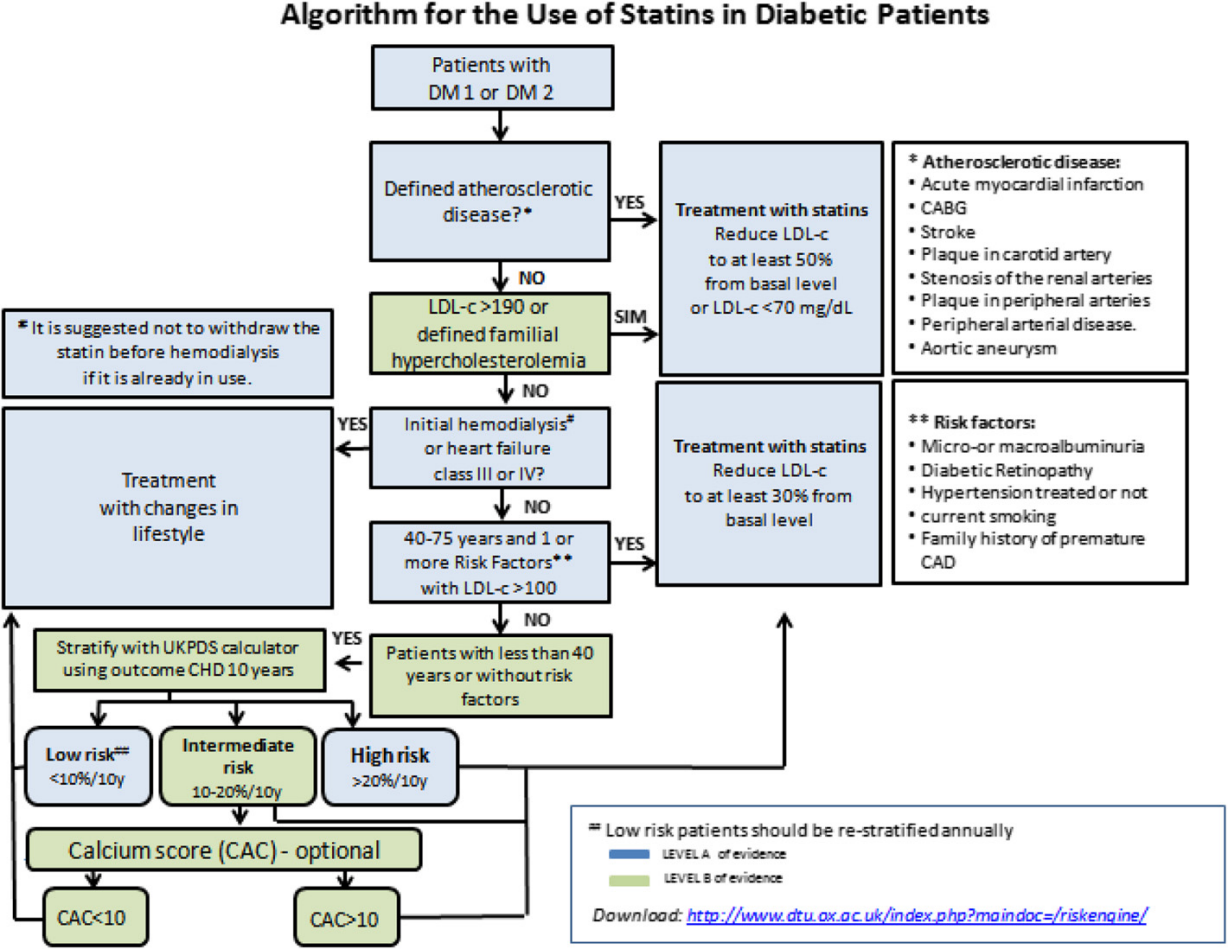
Decision making flow for statin use.

Based on the currently available literature and after careful review of national and international recommendations by the Expert Group on Diabetes and Cardiovascular Risk of the Brazilian Diabetes Society (SBD), the following algorithm for suggested criteria for statin therapy in patients with diabetes was developed (Figure 2).

### Module 4: hypertension

22. *** Patients with blood pressure levels greater than or equal to 140/90 mmHg should be considered hypertensive. Patients with levels close to these limits, with no evidence of organ damage, should be reevaluated periodically. It is recommended to measure blood pressure at each routine visit. [LEVEL A]***

Summary of evidence:

• *A meta-analysis of 61 observational studies*[47]*showed that, in the range of 40–69 years old, each increment of 20 mmHg systolic and 10 mmHg diastolic, is associated to a 2-fold increase in incidence in death from stroke and coronary ischemia. Blood pressure above 115/75 mmHg is associated with an increased risk for cardiovascular events, mortality and end stage renal disease in patients with diabetes*[47]*.*

23. *** It is recommended that patients with systolic blood pressure between 120–139 mmHg or diastolic pressure between 80–90 mmHg should be treated with non-pharmacological measures to control blood pressure. [LEVEL C]***

Summary of evidence:

• *Because there is no clear evidence of benefit from pharmacological treatment with systolic blood pressure below 140 mmHg, only recommended lifestyle modifications are indicated. A randomized clinical trial including 412 subjects*[48]*compared diets with high, medium and low sodium during 30 consecutive days. The DASH diet (low sodium) resulted in a reduction in average systolic blood pressure of 7.1 mmHg compared to the control diet in individuals without hypertension and of 11.5 mmHg in hypertensive individuals. The diet, however, has not been tested in patients with diabetes. Although there are no well-controlled study in the treatment of hypertension with diet and exercise in patients with diabetes studies, the DASH study group showed similar effects with pharmacological treatment in other studies, with results accepted for patients with diabetes, since the risk of adverse effects is negligible. It is recommended that sodium intake should be kept at less than 1,500 mg/day. Excess weight should be controlled. The use of low-fat dairy products, 2–3 daily servings of fruits and vegetables, limited alcohol intake and regular physical activity should be advised.*

24. *** It is recommended that patients with hypertension defined as systolic blood pressure equal to or greater than 140 mmHg or diastolic blood pressure greater than or equal to 90 mmHg receive pharmacological treatment. [LEVEL A]***

Summary of evidence:

• *A meta-analysis of 31 randomized controlled trials with 73,913 patients with diabetes*[49]*compared the effect of antihypertensive treatment regimens defined as intensive or less intensive in outcomes such as myocardial infarction and stroke. The meta-analysis found that risk-reductions for stroke and MI are firmly established when comparing antihypertensive agents against placebo. However, when comparing more intensive with less intensive regimen, there was no additional benefit, except a reduction in stroke. Protection is progressive for stroke, but not for acute myocardial infarction. However, no increased incidence of AMI is observed with more intensive blood pressure reductions, suggesting that reductions below to around 130 mmHg, can be safely pursued.*

25. *** It is recommended to reduce systolic pressure to below 140 mmHg and diastolic pressure to around 80 mmHg, regardless of the presence of micro or macrovascular complications. [LEVEL A]***

Summary of evidence:

• *The ACCORD BP study*[50]*was a non-blind arm of the ACCORD trial that directly compared the antihypertensive treatment goals with systolic BP <120 mmHg vs. Systolic BP <140 mmHg in 4,733 patients with type 2 diabetes at high cardiovascular risk, with follow-up of 4.7 years. The mean systolic BP was 119.3 mmHg in the intensive group and 133.5 mmHg in the conventional group. The primary composite outcome (nonfatal MI, nonfatal stroke, and cardiovascular death) was not different between groups. In prespecified secondary outcome, the incidence of stroke was lower in the intensive group (p <0.001). However, the intensive group had a higher rate of adverse effects such as hypotension, hypokalemia, and increased creatinine. Thus, in high-risk patients, it is recommended to reduce systolic BP below 140 mmHg but not below 120 mmHg due to the increased risk of adverse effects.*

• *A meta-analysis of 5 trials, both observational and randomized clinical trial, including 7,312 patients with diabetes showed that intensive treatment with a reduction in systolic BP to below 130 mmHg vs. reduction of systolic BP to below 140 mmHg showed reduction of the risk of stroke but not in mortality or in acute myocardial infarction*[51]*.*

26. *** Due to the similarity of benefit in cardiovascular outcomes, it is recommended that the decision in the choice of initial drug therapy, including the major classes of antihypertensive drugs, is based on tolerability, cost, and presence of comorbidities. Regarding renal protection, ACE inhibitors and angiotensin receptor blockers are superior. [LEVEL A]***

Summary of evidence:

• *A meta-analysis of 27 randomized trials with 158,709 patients including 33,395 patients with diabetes, the options of diuretics, calcium dihydropyridine blockers, ACE inhibitors and angiotensin II receptor blockers (ARBs) were equally effective in reducing cardiovascular events in subjects with or without diabetes*[52]*.*

• *A meta-analysis including 63 randomized controlled trials with 36,917 patients with diabetes compared different antihypertensive drugs versus placebo*[53]*. ACE inhibitors have consistently shown higher probability in being superior compared to other antihypertensive drugs in relation to renal outcomes, mortality from all causes, onset of dialysis or doubling of creatinine. The meta-analysis also demonstrated superiority of ACE inhibitors in relation to reno-protection, with similar benefit when ARBs are used.*

27. *** In the need of using more than one antihypertensive to achieve target blood pressure, it is preferably recommended the association between an angiotensin converting enzyme inhibitor with a dihydropiridin calcium blocker. [LEVEL A]***

Summary of evidence:

• *The randomized ACCOMPLISH trial*[54]*included 11,506 patients with high blood pressure and with cardiovascular high risk, including 60% of patients with diabetes. These patients were randomly assigned to associations to receive benazepril plus amlodipine or benazepril plus hydrochlorothiazide for 36 months. The study showed that the combination benazepril plus amlodipine is superior to the combination of benazepril plus hydrochlorothiazide in reducing the risk of cardiovascular events and mortality (reduction of relative risk of 19.6% p <0.001). Subgroup analysis showed that patients with diabetes had similar benefits as the non diabetic patients.*

• *In a meta-analysis comparing various strategies for antihypertensive treatment including 36,917 patients with diabetes, a total of 640 (1.7%) patients received combination ACEI-calcium blocker. The combination of ACEI- Calcium Channel Blocker was shown to have the most significant effect in reducing mortality compared to other combinations*[53]*.*

28. ***The use of ACE inhibitors or ARBs in patients with micro or macroalbuminuria is recommended. [LEVEL A]***

Summary of evidence:

• *In diabetic patients with macroalbuminuria the RENAAL study*[55]*showed efficacy of losartan compared to placebo in reducing renal outcomes as doubling creatinine, end- stage renal disease or death. Likewise, the IDNT study*[56]*demonstrated superiority of irbesartan compared to amlodipine in the reduction of the same renal endpoints.*

• *In patients with microalbuminuria the IRMA-2 study*[57]*showed progressive reduction of albuminuria with increasing doses of irbesartan.*

29. *** It is recommended to AVOID the combination of ACE inhibitors with ARBs due to greater risk of loss of renal function and hyperkalemia. [LEVEL A]***

Summary of evidence:

• *The ONTARGET study*[58]*evaluated the effects of adding an ACE inhibitor (ramipril) to an ARB (telmisartan) in 25,620 patients over 55 years, high-risk, including 9,603 with diabetes during 56 months. Participants were randomized into 3 groups: ramipril, telmisartan and a combination of ramipril with telmisartan. The primary renal outcome was a composite of dialysis, doubling of creatinine and death. The primary outcome was worse with the combination ramipril-telmisartan than with each of antihypertensive monotherapy (HR 1.09 95% CI: 1.01-1.18 p = 0.037). Thus, the ACEI-ARB combination since is contra-indicated due to its association with worsening of renal outcomes.*

30. *** In pursuit of the goals of blood pressure, other options of combination of 3 or more drugs may be considered, depending on clinical criteria, including ACEI or ARB with amlodipine, thiazide diuretics, beta-blockers, spironolactone, and vasodilators, but with a lower level of evidence. [LEVEL D]***

### Module 5: antiplatelet therapy

31. *** The use of acetylsalicylic acid (ASA) (75–162 mg/day) for coronary risk reduction in patients with diabetes and previous cardiovascular events is recommended. [LEVEL A]***

Summary of evidence:

• *Meta-analysis of the Antithrombotic Trialists (ATT) Collaboration*[59]*showed that in secondary prevention trials, aspirin compared to placebo resulted in significant reduction of major vascular events (6.7% vs 8.2% per year, p < 0,0001), with no increase in the risk of hemorrhagic stroke, but significant reduction in total stroke (2.08% vs 2.54% per year, p = 0.002) and coronary events (4.3% vs 5.3% per year p < 0.0001).*

• *The use of ASA for secondary cardiovascular prevention was associated with a significant reduction in mortality in a meta-analysis of 13 studies involving diabetic patients (RR: 0.82, 95% CI: 0.69 to 0.98, p = 0.03)*[60]*.*

32. *** In patients with diabetes without a history of cardiovascular events (primary prevention) ASA use could be considered only for high-risk patients (>20% in 10 years). For patients at low or moderate risk, ASA use is not usually recommended since the risk of significant gastrointestinal bleeding may outweight the cardiovascular benefit. [LEVEL A]***

Summary of evidence:

• *The effect of ASA was reported in only 2 large studies with diabetic patients. The POPADAD study*[60]*evaluated 176 patients with age above 40 years, with both DM1 and DM2, without prior cardiovascular disease. Patients were randomized to receive ASA 100 mg, antioxidant, antioxidant plus ASA or placebo for 5 years. There was no difference in the primary outcome between the groups.*

• *In the second study, JPAD study*[61]*, researchers examined the efficacy of aspirin in primary prevention in an open study in 2,539 Japanese individuals with diabetes without a history of cardiovascular disease. Patients were randomized to ASA or nothing and were followed for 4.4 years. There was no difference in the total number of events between the group receiving ASA and those who did not receive it (HR 0.80 95% 0.58 to 1.10). The secondary outcome of coronary and cerebrovascular mortality however, was favorable to the ASA group (HR 0.10 95% 0.01 to 0.79).*

• *A meta-analysis by the Trialists (ATT)*[59]*including 6 large studies with ASA in primary prevention in the general population including 95,000 individuals and 4,000 with diabetes, showed that ASA reduces by 12% (RR 0.88, 95% 0.82 to 0.94) the risk of vascular events, with the largest reduction for nonfatal MI, with little effect on coronary death and a relative increase in the risk of hemorrhagic stroke (32%). The effect was similar in the subgroup of patients with diabetes.*

• *In another meta-analysis*[62]*only with diabetic patients, including POPADAD and JPAD studies, showed that ASA was associated with non-significant 9% reduction in risk of coronary events and 10% in stroke.*

• *A meta-analysis that evaluated the use of low-dose aspirin in 9 studies (observational and randomized clinical trials) in 11,787 diabetic patients also showed no significant benefit (9% reduction) in relation to coronary events (95% CI 0.79 - 1.05) as compared to AVC (non-significant reduction of 10% (95% CI 0.71-1.13)*[63]*. Similar results were found in 2 other meta-analysis*[64,65]*. In another meta-analysis in general population*[64]*, ASA reduced risk of MI only in men (relative risk, 0.57 [95% CI 0.34 to 0.94]).*

• *The absolute risk of hemorrhagic stroke with low-dose aspirin in the general population is 1:10,000 people annually*[66]*. In relation to gastro-intestinal bleeding, the absolute risk of gastrointestinal bleeding with aspirin in middle-aged adults is 3 per 1000 per year*[63]*. Patients with diabetes have a relative increase of 55% in the risk of bleeding (95% CI 1.113 to 2.14) compared to patients without diabetes, observed in meta-analysis of 6 studies by Trialists in primary prevention*[59]*. The combined use of proton pump blockers can reduce this risk*[67]*.*

33. *** In patients with defined cardiovascular disease and documented allergy to ASA, clopidogrel may be used. [LEVEL C]***

Summary of evidence:

• *The CAPRIE study*[68]*was a randomized double-blind clinical trial that compared the relative efficacy of clopidogrel 75 mg compared to ASA 325 mg in reducing the primary composite end point of ischemic stroke, myocardial infarction or vascular death in patients with ischemic stroke, r acute myocardial infarction and recent peripheral vascular insufficiency for a follow-up of 1–3 years. There were 19,185 patients included being 20% of diabetic patients. The study showed superiority of clopidogrel compared with ASA in preventing the primary outcome (Relative risk reduction of 8.7% in favour of clopidogrel (95% CI 0.3-16.5) with a similar safety profile. Data for diabetic patiets are indirect.*

34. *** In patients with diabetes presenting with acute coronary syndrome (ACS) it is recommended the dual antiplatelet treatment with aspirin associated with a P2Y12 receptor antagonist for 1 year after the acute event. Data from clinical trials indicate that the benefit is greater with prasugrel and ticagrelor compared to clopidogrel in the general population with ACS. In a subgroup analysis of patients with diabetes, prasugrel was superior to clopidogrel in patients with ACS undergoing PCI. [LEVEL B]***

Summary of evidence:

• *The results of five randomized trials that compared the combination of ASA plus of P2Y12 receptor antagonist with ASA alone are consistent in showing greater benefit in reducing cardiovascular outcomes with the combination. The studies, however, were not performed exclusively in patients with diabetes. Thus, there is a limitation for the interpretation of these results. However, this committee recommends the use of this combination in patients with diabetes. The evidence supports the use of clopidogrel and ticagrelor when no percutaneous coronary intervention (PCI) is performed, and clopidogrel, prasugrel or ticagrelor when PCI is performed*[69-73]*.*

• *The TRITON-TIMI 38 study compared prasugrel with clopidogrel in 13,608 patients using aspirin after percutaneous intervention. A prespecified subgroup analysis in 3,146 patients with diabetes showed a reduction of 30% (HR 0.7 95% CI 0.58 to 0.85, p < 0.001) in the primary endpoint with prasugrel compared to clopidogrel. No significant interaction effect between the treatment and the presence of diabetes, indicating that the data can be extended to diabetic patients*[73]*. As this is a result of a substudy these results are yet to be confirmed for patients with diabetes.*

### Module 6: myocardial revascularization

35. *** It is recommended to consider coronary artery bypass graft (CABG) in the following situations [LEVEL B]:***

– ***Myocardial ischemia symptoms not controlled by medical treatment.***

– ***Suspected extensive myocardial ischemia.***

– ***Suspected ischemia with left ventricular dysfunction and myocardial viability.***

– ***Obstruction of greater than 50% in the left main coronary artery.***

Summary of evidence:

• *The objectives of myocardial revascularization (MR) are to reduce long-term mortality and improve symptoms and quality of life in patients not well controlled with optimal medical therapy. Patients with diabetes and coronary artery disease have higher long-term mortality compared to patients without diabetes, regardless of treatment strategy*[74]*. Just as for patients without diabetes, the best criterion for decision MR in patients with diabetes is the clinical assessment, considering the presence of myocardial ischemia (symptoms or evidence of complementary tests), evaluation of coronary anatomy and left ventricular systolic function. Indications for MR in patients with diabetes are similar to those for patients without diabetes, respecting the existing guidelines*[75-77]*.*

36. *** Clinical treatment is recommended as the initial strategy in cases of chronic stable coronary artery disease with preserved ventricular function, controlled symptoms, evidence of myocardial ischemia and coronary anatomy without high-risk criteria. [LEVEL B]***

Summary of evidence:

• *The COURAGE study*[78]*randomized 2,287 patients with chronic CAD to receive optimal medical therapy or angioplasty with stent associated with clinical treatment. In the subgroup of 766 patients with diabetes there was no difference between groups with respect to the combined endpoint of mortality and nonfatal myocardial infarction (HR: 0.99, 95% CI 0.73 to 1.32). Importantly, patients with more severe angina functional class IV (CCS IV), heart failure (HF), ejection fraction (LVEF) <30%, recent myocardial revascularization and evidence of ischemia at high risk were excluded from the study.*

• *In BARI 2D study*[79,80]*authors randomized 2,368 patients with type 2 diabetes and chronic coronary artery disease to initial strategy of optimal medical therapy or medical treatment with percutaneous or surgical revascularization. There was no difference between groups in relation to survival in a follow-up of 5 years (87.8% in the medical therapy group vs. 88.3% in the revascularized group, p = 0.97). However, in patients undergoing surgical revascularization, there was a lower rate of cardiovascular events (death, MI, or stroke) compared to patients in the clinical treatment (22.4% vs 30.5%, p = 0.01, respectively). This study also excluded patients with lesions of the left main coronary artery and HF patients.*

37. *** In patients with obstruction in more than one arterial territory with indication for CABG, surgery should be the preferred strategy in relation to angioplasty, provided that the anatomy is favorable to the surgical procedure. [LEVEL A]***

Summary of evidence:

• *The BARI study compared surgical Myocardial Revascularization with balloon angioplasty in patients with chronic coronary artery disease, and in the subgroup of patients with diabetes, survival was higher in the surgical group after a 5 to 10 years follow-up*[79,80]*.*

• *A meta-analysis using individual data from 7,812 patients showed a 30% reduction in mortality in the subgroup of 1,233 patients with diabetes undergoing CABG*[81]*.*

• *The SYNTAX study subdivided the groups in relation to the anatomical complexity, developing the SYNTAX score*[82]*. On this score, patients with low anatomical complexity (SYNTAX score ≤22) had the same morbidity and mortality during follow-up. However, in the subgroup of patients with anatomically complex lesions (high SYNTAX score ≥33) showed a higher mortality with angioplasty compared to surgery (13.5% vs 4.1%, p = 0.04, respectively)*[82]*. In the 5 years follow-up these patients confirmed the greater number of cardiovascular events in the angioplasty group (46.5% vs. 29%; p < 0.001) in patients with high SYNTAX*[83]*score. In this analysis there was a trend toward lower mortality in the CABG group, but no statistically significant difference (p = 0.065)*[84]*.*

• *A subanalysis of the same study (SYNTAX)*[82]*including 452 patients with diabetes, in which it was compared surgery vs. stent angioplasty, patients undergoing angioplasty with pharmacological stent and lesion of the left main coronary artery or three-vessel disease presented a higher rate of cardiovascular events at one year than those who underwent surgical revascularization (26% vs. 14.2% respectively, p = 0.003).*

• *The FREEDOM study*[85]*randomized 1,900 patients with diabetes and at least bi-arterial lesions (excluding injury in left main coronary artery trunk) for surgical or percutaneous revascularization with stent drug (sirolimus and paclitaxel). After five years follow-up, patients who underwent surgery had lower overall mortality (10.9 vs 16.3%; p = 0.049, respectively) and lower incidence of AMI (6 vs. 13%, 9%, p < 0.001) than those who underwent angioplasty. It should be emphasized that these patients were not subdivided in relation to its anatomical complexity*[85]*. Therefore, for multi-arterial patients with indication for CABG, surgery should be the preferred strategy in relation to angioplasty, provided the anatomy is favorable to the surgical procedure and the surgical risk be acceptable.*

38. *** In patients with diabetes and clinical indication for percutaneous myocardial revascularization, we suggest the use of pharmacological stent, provided there is no contraindication to double therapy of anti-platelet aggregation for a minimum period of one year. [LEVEL B]***

Summary of evidence:

• *Patients with diabetes treated with drug-eluting stents have a lower rate of need for repeat revascularization than patients treated with non-drug-eluting stents*[86,87]*. Following five years of ARTS I and II studies*[85,88]*, patients with diabetes undergoing angioplasty with drug eluting stents with sirolimus had a lower rate of AMI and less need for revascularization than patients who received non-pharmacological stent (4,8% vs. 11%; 0.04 and p = 33.2% vs. 43.7%; p = 0.02, respectively).*

## Competing interests

Marcello Casaccia Bertoluci, The author declares no competing interest. Augusto Pimazoni-Netto, Sanofi, Becton, Dickinson and Company. Antonio Carlos Pires, Sanofi, BMS, Astra Zeneca, MSD. Antonio Eduardo Pesaro, The author declares no competing interest. Beatriz D. Schaan, The author declares no competing interest. Bruno Caramelli, The author declares no competing interest. Carisi Anne Polanczyk, The author declares no competing interest. Carlos Vicente Serrano Júnior, Novartis, MSD, Boehringer. Danielle M. Gualandro, The author declares no competing interest. Domingos Augusto Malerbi, Sanofi. Emilio Moriguchi, Pfizer, Biolab, Daiichi-Sankyo, MSD. Flavio Antonio de Oliveira Borelli, The author declares no competing interest. João Eduardo Nunes Salles, Aché, Abbott, Astra Zeneca, BMS, Lilly, Jansen, Novartis, Sanofi. José Mariani Júnior, The author declares no competing interest. Luis Eduardo Rohde, The author declares no competing interest. Luis H. Canani, Abbott, BMS, Lylly, MSD, Novo Nordisk, Pfizer, Sanofi, MannKind, GSK, Roche, Boehringer. Luiz Antonio Machado Cesar, Servier. Marcos Tambascia, The author declares no competing interest. Maria Tereza Zanella, Novo Nordisk, Boehringer, Abbott, Astra Zeneca. Miguel Gus, The author declares no competing interest. Rafael Selbach Scheffel, The author declares no competing interest. Raul Dias dos Santos, Amgen, Aegerion, Astra Zeneca, Boehringer, BMS, Biolab, Genzyme, Pfizer, Novo-Nordisk, Novartis, MSD, Sanofi/Regeneron and Nestle.

## Authors’ contributions

All authors had full participation in the search for references, in the development of the contents of this Position Statement and also in the peer review procedure of the final text. MCB^1^ acted as the Chief Editor. APN^2^ was responsible for the English translation and for the overall editorial coordination. All authors read and approved the final manuscript.
